# Phenotypic analysis of mutations at residue 146 provides insights into the relationship between NS5A hyperphosphorylation and hepatitis C virus genome replication

**DOI:** 10.1099/jgv.0.001366

**Published:** 2019-12-10

**Authors:** Niluka Goonawardane, Chunhong Yin, Mark Harris

**Affiliations:** ^1^​ School of Molecular and Cellular Biology, Faculty of Biological Sciences and Astbury Centre for Structural Molecular Biology, University of Leeds, Leeds, LS2 9JT, UK; ^†^​ Present address: Experimental Medicine, Nuffield Department of Medicine, The Peter Medawar Building for Pathogen Research, South Parks Road, University of Oxford, Oxford, OX1 3SY, UK; ^‡^​ Present address: Tsinghua-Peking Center for Life Sciences, School of Medicine, Tsinghua University, Beijing 100084, PR China

**Keywords:** hepatitis C virus, NS5A, phosphorylation, RNA replication, sub-genomic replicons

## Abstract

The hepatitis C virus genotype 2a isolate, JFH-1, exhibits much more efficient genome replication than other isolates. Although basic replication mechanisms must be conserved, this raises the question of whether the regulation of replication might exhibit isolate- and/or genotype-specific characteristics. Exemplifying this, the phenotype of NS5A hyperphosphorylation is genotype-dependent; in genotype 1b a loss of hyperphosphorylation correlates with an enhancement of replication. In contrast, the replication of JFH-1 is not regulated by hyperphosphorylation. We previously identified a novel phosphorylation site in JFH-1 NS5A: S146. A phosphomimetic substitution (S146D) had no effect on replication but correlated with a loss of hyperphosphorylation. In genotype 1b, residue 146 is alanine and we therefore investigated whether the substitution of A146 with a phosphorylatable (S), or phosphomimetic, residue would recapitulate the JFH-1 phenotype, decoupling hyperphosphorylation from replication. This was not the case, as A146D exhibited both a loss of hyperphosphorylation and a reduction in replication, accompanied by a perinuclear restriction of replication complexes, reductions in lipid droplet and PI4P lipid accumulation, and a disruption of NS5A dimerization. In contrast, the S232I culture-adaptive mutation in the low-complexity sequence I (LCSI) also exhibited a loss of hyperphosphorylation, but was associated with an increase in replication. Taken together, these data imply that hyperphosphorylation does not directly regulate replication. In contrast, the loss of hyperphosphorylation is a consequence of perturbing genome replication and NS5A function. Furthermore, we show that mutations in either domain I or LCSI of NS5A can disrupt hyperphosphorylation, demonstrating that multiple parameters influence the phosphorylation status of NS5A.

## Introduction

Hepatitis C virus (HCV) is a leading cause of liver disease worldwide, with an estimated 71 million chronic infections that can ultimately lead to liver fibrosis, cirrhosis or hepatocellular carcinoma [1]. HCV belongs to the family *Flaviviridae*, enveloped viruses with a positive-sense RNA genome (9.6 kb) coding for a single polyprotein that is processed co- and post-translationally by viral and host proteases, yielding four structural proteins (core, E1, E2 and p7) and six non-structural proteins (NS2, NS3, NS4A, NS4B, NS5A and NS5B) [2].

**Fig. 1.**
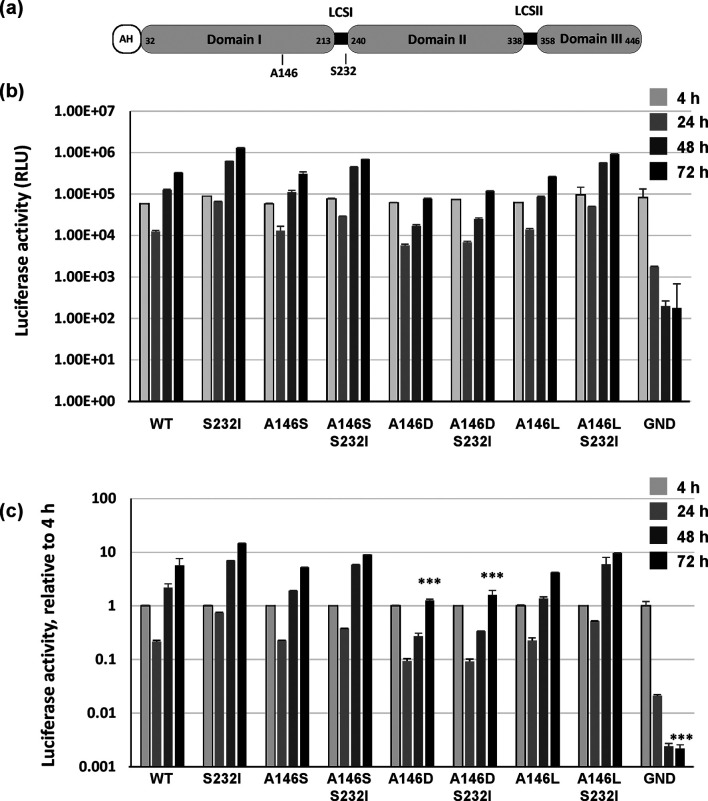
Role of NS5A residue 146 in genotype 1b RNA replication. (a) Schematic of NS5A structure showing amino acid residue numbers for Con1 and the location of A146 and S232. AH, amphipathic helix, LCS, low-complexity sequence. (b, c) Huh7.5 cells (4.3×10^6^) were electroporated with *in vitro* transcripts of either wild-type Con1 SGR (CpG/UpA-low luciferase), NS5A mutants (A146S, A146L, A146D) with or without S232I substitution, or the NS5B GND mutant, seeded into 96-well plates and incubated for 4, 24, 48 and 72 h post-electroporation (p.e.). Absolute values of firefly luciferase activity are shown (b), together with values normalized to the 4 h p.e. reading (c). Error bars: sem, data from three independent experiments are shown. Significant differences from the wild-type are denoted by *** (*P*<0.001).

Recently, virus genome replication has become the target for a number of potent direct-acting antivirals (DAAs) that have revolutionized therapy for HCV infection. These small molecule inhibitors are targeted directly to the NS3 protease, NS5B polymerase and NS5A. Although the mode of action of NS3 and NS5B inhibitors is well defined, it is not clear how NS5A inhibitors such as daclatasvir (DCV) function. NS5A is a phosphoprotein that has multiple functions in virus genome replication, assembly and modulation of host cell biology [3]. NS5A comprises three distinct domains (I–III), separated by short low-complexity sequences (LCSI and II) [4] (Fig. 1a). Three structural analyses of the well-conserved domain I revealed similar monomer structures that adopt different dimer conformations [5–7]. NS5A is highly phosphorylated [8], and two species with differing migration on SDS-PAGE are designated p56 (basal) and p58 (hyper). Phosphorylation of NS5A appears to be a highly regulated process and several cellular kinases such as Polo-like kinase 1 (Plk1) [9] and casein kinase Iα (CKIα) [10, 11] have been implicated in hyperphosphorylation within the serine-rich LCSI. Phosphatidylinositol 4-kinase III alpha (PI4K-IIIα) [12] and CKII [13] have been implicated in basal phosphorylation, mainly in domains II/III [14, 15]; however, as yet, neither the kinases that phosphorylate NS5A nor the amino acid residues that are phosphorylated, have been unambiguously defined.

The first stage in addressing these important questions was to define the phosphorylation sites within NS5A. To this end, we [8], and others [10, 11, 16–18], used mass spectrometric approaches. In our study we showed that in the context of a subgenomic replicon (SGR), JFH-1 NS5A was phosphorylated on multiple serine residues, predominantly within LCSI. Mutagenesis revealed a role for a subset of these phosphorylation events in controlling genome replication [8]. The cluster of phosphorylation sites in LCSI was expected, as this had previously been described indirectly by mutagenesis [15], as well as by mass spectrometry [16]. Indeed, subsequent studies confirmed that LCSI is a major site for phosphorylation [10, 11, 17]. However, intriguingly, our analysis also identified a previously uncharacterized phosphorylated serine within domain I: S146 (Fig. 1a), corresponding to residue S2122 in the JFH-1 polyprotein (accession number AB047639). This residue was also identified independently in cells transfected with a non-replicating NS3-5B expressing plasmid [18]. In both cases, mutagenesis of S146 had no effect on genome replication, but a phosphomimetic aspartate substitution (S146D) inhibited hyperphosphorylation. As S146 is ∼75 residues N-terminal to LCSI, this observation implied that there was some crosstalk between domain I and LCSI to allow the regulation of hyperphosphorylation, for example the addition of a negative charge at residue 146 might result in a conformational change preventing hyperphosphorylation.

Interestingly, S146 is only present in genotype 1a and 2a isolates, and in the majority of other HCV isolates, including the prototype genotype 1b Con1 isolate, residue 146 is an alanine. The impact of residue 146 on the phosphorylation status of NS5A might therefore be exclusive to genotype 2a, and not a feature conserved throughout all HCV genotypes. In this context, the fact that the S146D-mediated inhibition of hyperphosphorylation did not affect JFH-1 genome replication contrasts with the situation for genotype 1b, where inhibition of hyperphosphorylation, for example as associated with the highly culture-adaptive S232I mutation within LCSI (polyprotein numbering S2204I in the genotype 1b Con1 isolate, accession number Q9WMX2), enhanced replication [19–21]. This points to the conclusion that the factors affecting the phosphorylation status of NS5A differ between HCV genotypes.

In this study we sought to address this question by investigating the role of residue 146 in the context of a genotype 1b isolate (the prototypic Con1 strain) [22]. To do this we mutated this residue in a Con1 SGR to generate a phosphorylatable residue (A146S), or phosphomimetic (A146D), and compared the phenotype with the previously characterized culture-adaptive mutant S232I. Both S232I and A146D reduced NS5A hyperphosphorylation. However, whereas S232I exhibited the expected enhancement of genome replication, A146D reduced replication and altered the distribution of NS5A and other replication complex markers, restricting them to a perinuclear region. These data are consistent with the hypothesis that hyperphosphorylation of NS5A is dependent on multiple factors and in genotype 1b is not directly correlated with genome replicative capacity. In addition, we propose that both the protein structure and regulation of NS5A function differ between different genotypes of HCV and that caution must be exercised in extrapolating data obtained from one isolate of the virus. In particular, JFH-1 may not be representative of other genotypes.

## Results

### Role of residue 146 in Con1 genome replication and NS5A hyperphosphorylation

We had previously shown that hyperphosphorylation of JFH-1 NS5A was inhibited by a phosphomimetic mutation at residue S146 in domain I, in the absence of any concomitant effect on genome replication [8]. This contrasted with the situation in genotype 1b, where it had been previously reported that a loss of hyperphosphorylation correlated with enhanced replication [19–21], leading to the hypothesis that hyperphosphorylation negatively regulated genome replication. However, this observation in genotype 1b was exemplified by a culture-adaptive mutation, S232I, within LCSI, which presumably acted by directly disrupting the proposed hierarchical phosphorylation within this serine-rich sequence that has been associated with the hyperphosphorylated species. We therefore set out to ask whether residue 146 could also contribute to the regulation of hyperphosphorylation and/or replication in genotype 1b, with a view to shedding more light on both the mechanisms and consequences of hyperphosphorylation. We mutated A146 (A2119 in polyprotein numbering) in the genotype 1b consensus (Con1) SGR to either serine or aspartate (A146S and A146D, respectively), and to control for potential phosphorylation-independent effects of residue 146, it was also mutated to leucine (A146L). To facilitate a comparative analysis of the phenotype of these mutants, we also generated the previously characterized culture adaptive mutation S232I (S2204I in polyprotein numbering), previously shown to abrogate hyperphosphorylation and increase SGR replication <20 000 fold [19]. Lastly, to assess any potential synergistic or anergistic effects of these mutants, we combined each of the three A146 mutants with S232I. In order to avoid the potential confounding effects of selection (which might result in additional mutations) we set out to perform these studies in a transient replication system. For this reason, we mutated the NS5A sequence in a Con1 SGR that contained a modified firefly luciferase gene in which all CpG dinucleotides, and as many UpA dinucleotides as possible, were eliminated whilst retaining identical coding (kindly provided by Peter Simmonds, University of Oxford). This had been shown to result in a 100-fold enhancement of the replicative capacity of the Con1 SGR [23], comparable to the replication enhancement achieved in a similarly modified replicon for echovirus 7 [24], although the mechanism of this enhancement remains to be determined.

*In vitro* transcripts of the various mutant SGRs were electroporated into Huh7.5 cells and genome replication was followed over 72 h by assaying luciferase activity (Fig. 1b) and normalized to the 4 h post-electroporation (p.e.) value to assess transfection efficiency and the translation of input RNA (Fig. 1c). All mutants were able to replicate, but a variety of phenotypes were observed: whereas A146D resulted in about a 1-log reduction in genome replication efficiency, replication of A146S was similar to that of the wild-type. This was in contrast to the results previously observed for JFH-1, where mutation of S146 to either A or D had no effect on SGR replication or production of infectious virus [8]. The A146L mutation also had no effect on the level of genome replication. The addition of the S232I mutation significantly enhanced replication of the wild-type, A146S and A146L mutants, but only by <10-fold. This is considerably less than the original observation [19], but perhaps reflects the fact that the low CpG/UpA luciferase had already enhanced replication by approximately 100-fold. Interestingly, S232I did not exhibit a similar enhancement of the replication of the phosphomimetic A146D mutant, suggesting that the phenotype of A146D was dominant.

**Fig. 2.**
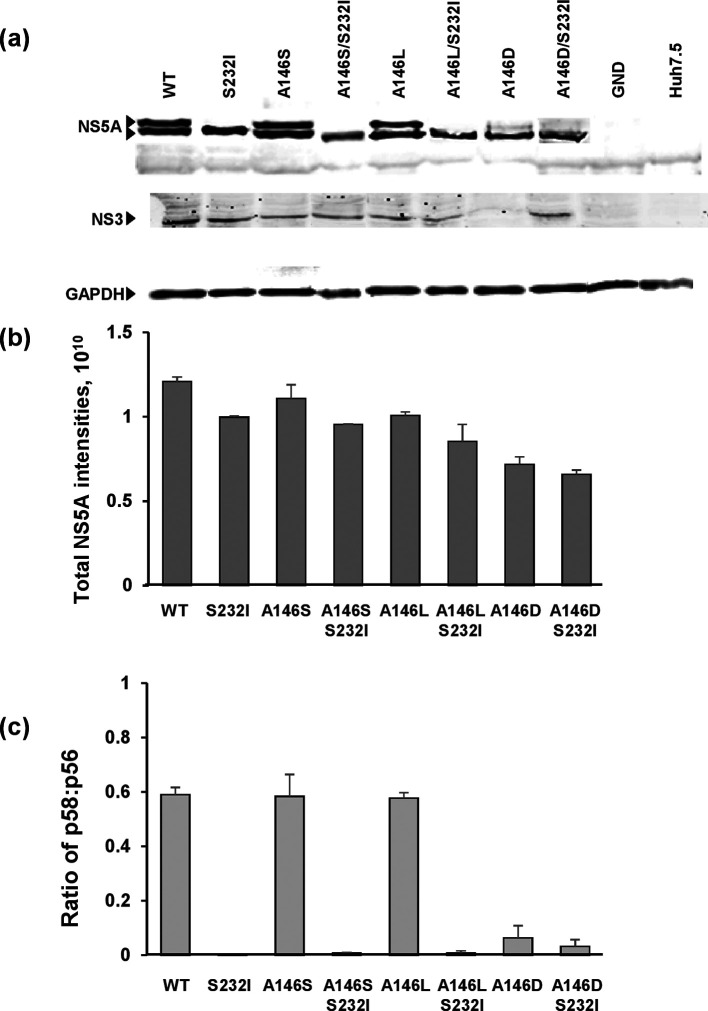
Effect of A146 mutation on NS5A expression and phosphorylation. Huh7.5 cells were electroporated with *in vitro* transcripts of the indicated SGR and incubated for 48 h p.e. (a) Cells were lysed and analysed by SDS-PAGE/Western blot with a sheep polyclonal antiserum to NS5A [34] or mouse monoclonal antiserum to NS3. (b, c) Total NS5A levels and the ratio of hyper- to basal-phosphorylation were quantified from fluorescent Western blots. Error bars show sem; data from three independent experiments are shown.

In the context of the JFH-1 SGR, the phosphomimetic S146D mutation resulted in a reduction in hyperphosphorylation [8, 25]. Our data showed that in Con1 the A146D mutation, which would impart a negative charge at this position, was deleterious to genome replication. We thus proceeded to investigate this phenotype in more detail to determine whether it was also associated with alterations to NS5A hyperphosphorylation. To assess this, Huh7.5 cells electroporated with Con1 SGRs were lysed at 48 h p.e. and analysed by SDS-PAGE/Western blotting (Fig. 2a). Total expression levels of NS5A normalized to GADPH, together with the ratio of hyper : basal-phosphorylated species, were determined (Fig. 2b, c). As expected, the wild-type SGR exhibited the characteristic two species of NS5A: p58 (hyperphosphorylated) and p56 (basal-phosphorylated). Neither the A146S nor the A146L mutations had any effect on the levels of NS5A expression or the p58 : p56 ratio. As seen for JFH-1, the phosphomimetic mutant A146D resulted in a significant reduction in NS5A hyperphosphorylation (Fig. 2a), and a modest reduction in the overall levels of NS5A expression. Furthermore, as previously demonstrated [19], the S232I substitution resulted in a complete loss of p58 – this was seen for the wild-type and all three A146 substitutions. These data show that a loss of NS5A hyperphosphorylation can be mediated via distinct mechanisms, and can correlate with either an enhancement (S232I) or the inhibition (A146D) of genome replication.

### The A146D mutation perturbs replication complex formation

**Fig. 3.**
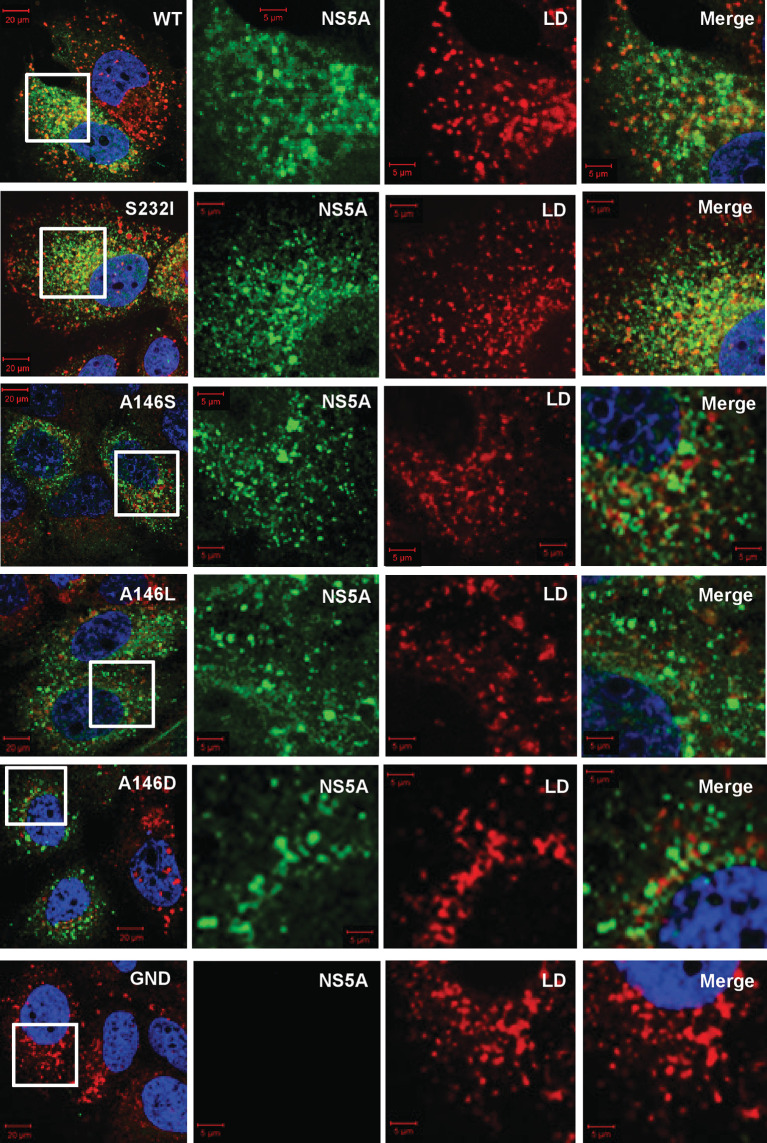
Sub-cellular distribution of NS5A and lipid droplets (LDs) in Con1 SGR electroporated cells. Huh7.5 cells were electroporated with *in vitro* transcripts of the indicated SGRs, and the cells were seeded onto coverslips and incubated for 48 h prior to fixation. Cells were subsequently permeabilized and immunostained for NS5A (sheep anti-NS5A), with LD detection using BODIPY(558/568)-C12 dye, prior to imaging by confocal microscopy. White boxes indicate the area expanded in the right-hand panels.

**Fig. 4.**
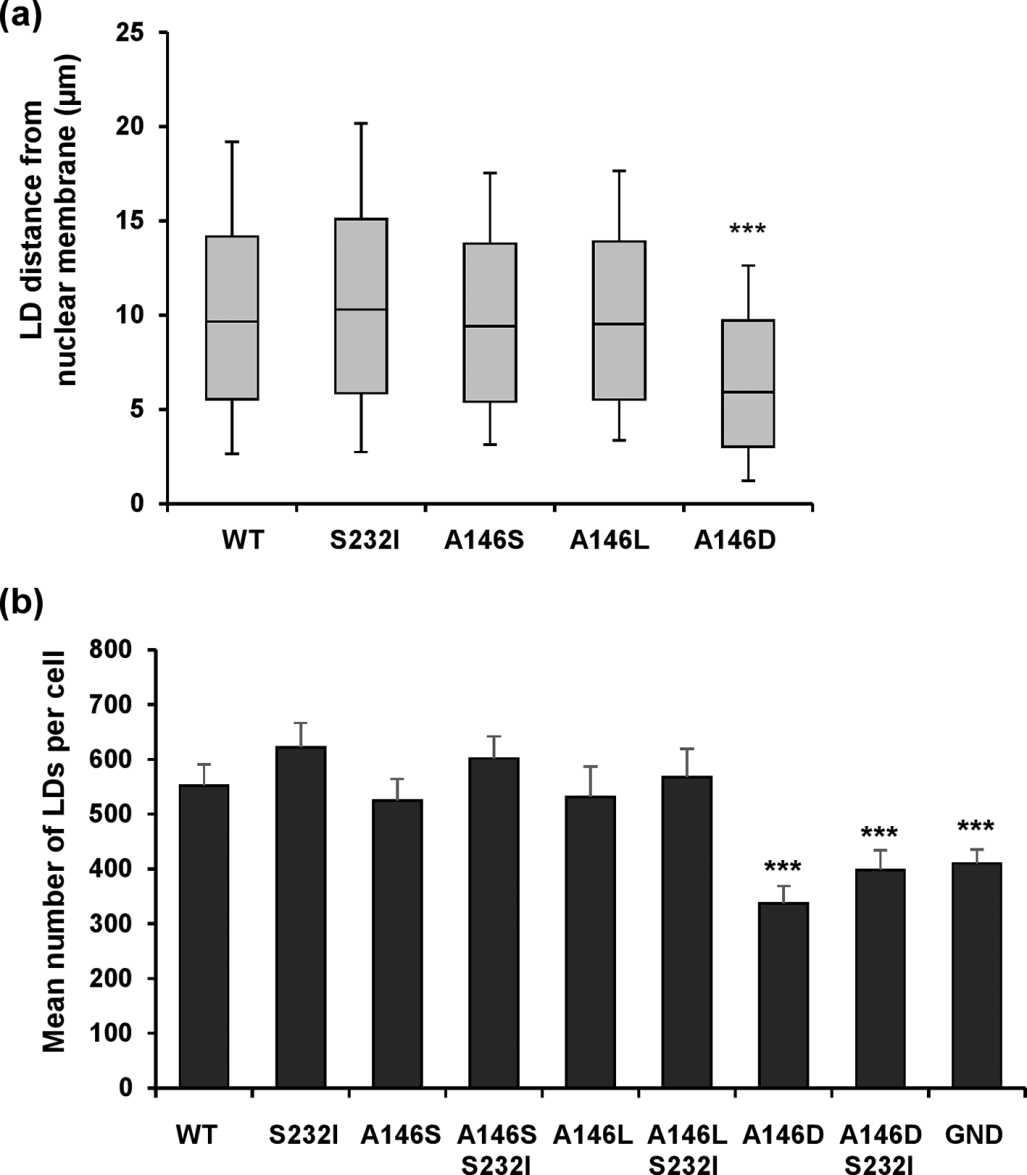
Quantification of lipid droplet (LD) distribution and abundance. (a) Spatial data for LDs were determined from 12 cells for each SGR using the GDSC plugin for Fiji. Box-and-whisker plots show the 10–90 % distribution for distance from the nuclear membrane. (b) Quantification of the total number of LDs per cell (c). Significant differences from the wild-type are denoted by *** (*P*<0.001).

To further analyse the consequences of a loss of NS5A hyperphosphorylation, we examined the sub-cellular distribution of components of the replication complex [NS3, NS5A and phosphatidylinositol-4-phosphate (PI4P) lipids] by confocal microscopy. We had previously demonstrated that in cells harbouring the wild-type JFH-1 SGR or infected with JFH-1, NS5A was observed throughout the cytoplasm in discrete punctae. Whereas some phosphorylation site mutants, exemplified by S225A, resulted in a restricted perinuclear distribution of NS5A and other replication complex components which correlated with a reduction in replication efficiency, S146A or S146D retained a wild-type distribution [25]. We therefore wished to test whether the Con1 A146D mutation, which was associated with a reduction in replication, also exhibited a restricted distribution. Huh7.5 cells were electroporated with the SGR panel, prior to fixation and detection of NS5A by immunofluorescence and BODIPY staining for lipid droplets (LDs) (Fig. 3), as we had previously noted a juxtaposition of NS5A and LDs. The wild-type and all mutants except A146D exhibited a broad cytoplasmic distribution of bright punctate NS5A staining, juxtapositioned with LDs. The distribution of both NS5A and LDs in the case of A146D was restricted to a perinuclear region, although this restricted distribution was not as pronounced as that observed previously for JFH-1 S225A [25]. This was quantified by determining LD spatial distribution data for 12 randomly selected cells, confirming that A146D exhibited a clustering of LDs close to the nucleus in comparison to the wild-type and the other mutants (Fig. 4a). The total number of LDs per cell was also determined. As expected, there was a significant increase in LDs for the wild-type SGR compared to GND, and a reduced abundance in the A146D mutant compared to the wild-type (Fig. 4b). In contrast, the number of LDs in the A146S and A146L mutants was broadly comparable to that in the wild-type. The S232I mutant exhibited a modest increase in LD numbers, but this did not reach statistical significance.

**Fig. 5.**
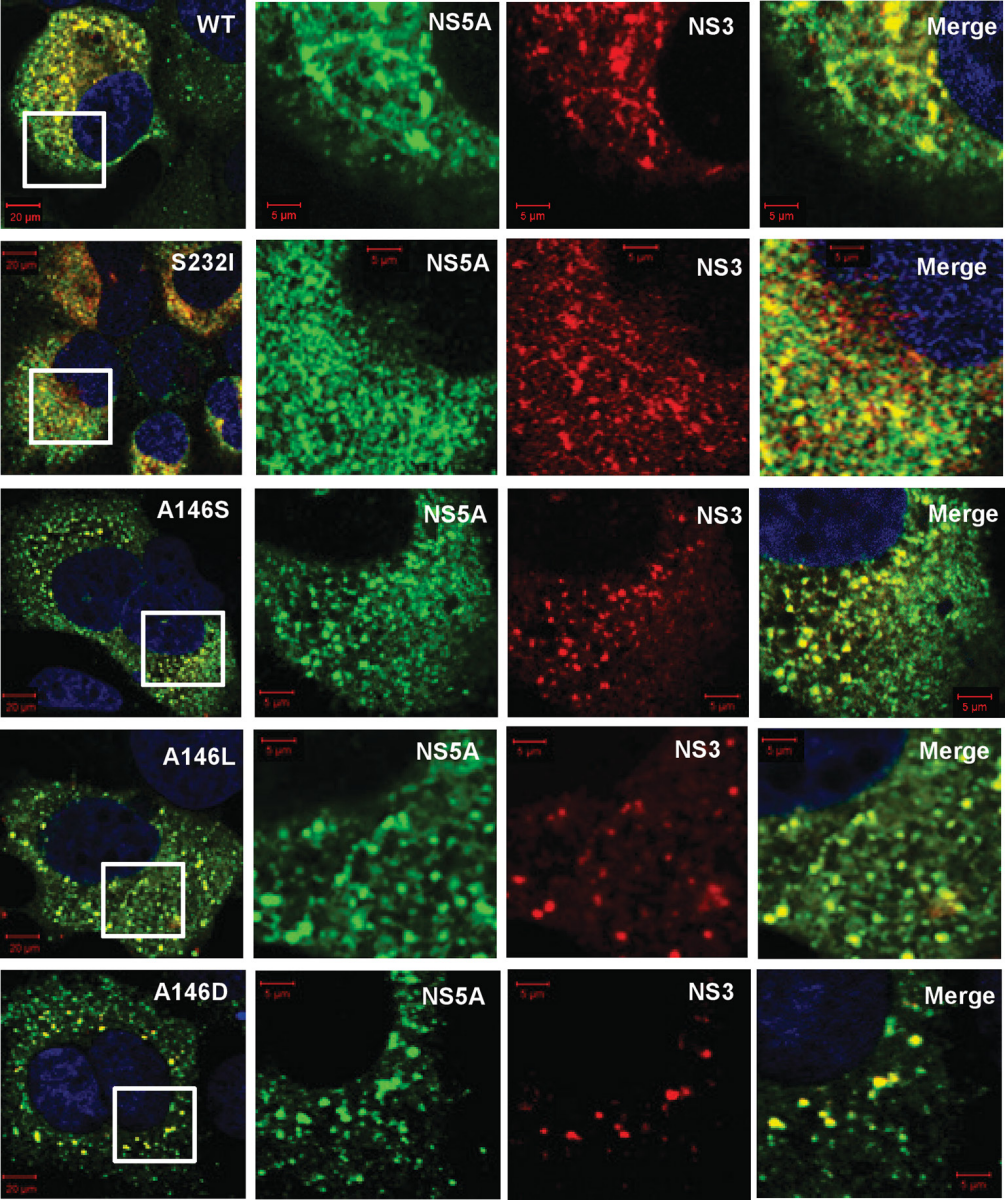
Colocalization of NS5A and NS3. Huh7 cells were electroporated with *in vitro* transcripts of the indicated SGRs, and seeded onto coverslips. At 48 h p.e. cells were fixed and immunostained for NS5A and NS3 (mouse monoclonal anti-NS3), prior to imaging by confocal microscopy. White boxes indicate the area expanded in the right-hand panels.

**Fig. 6.**
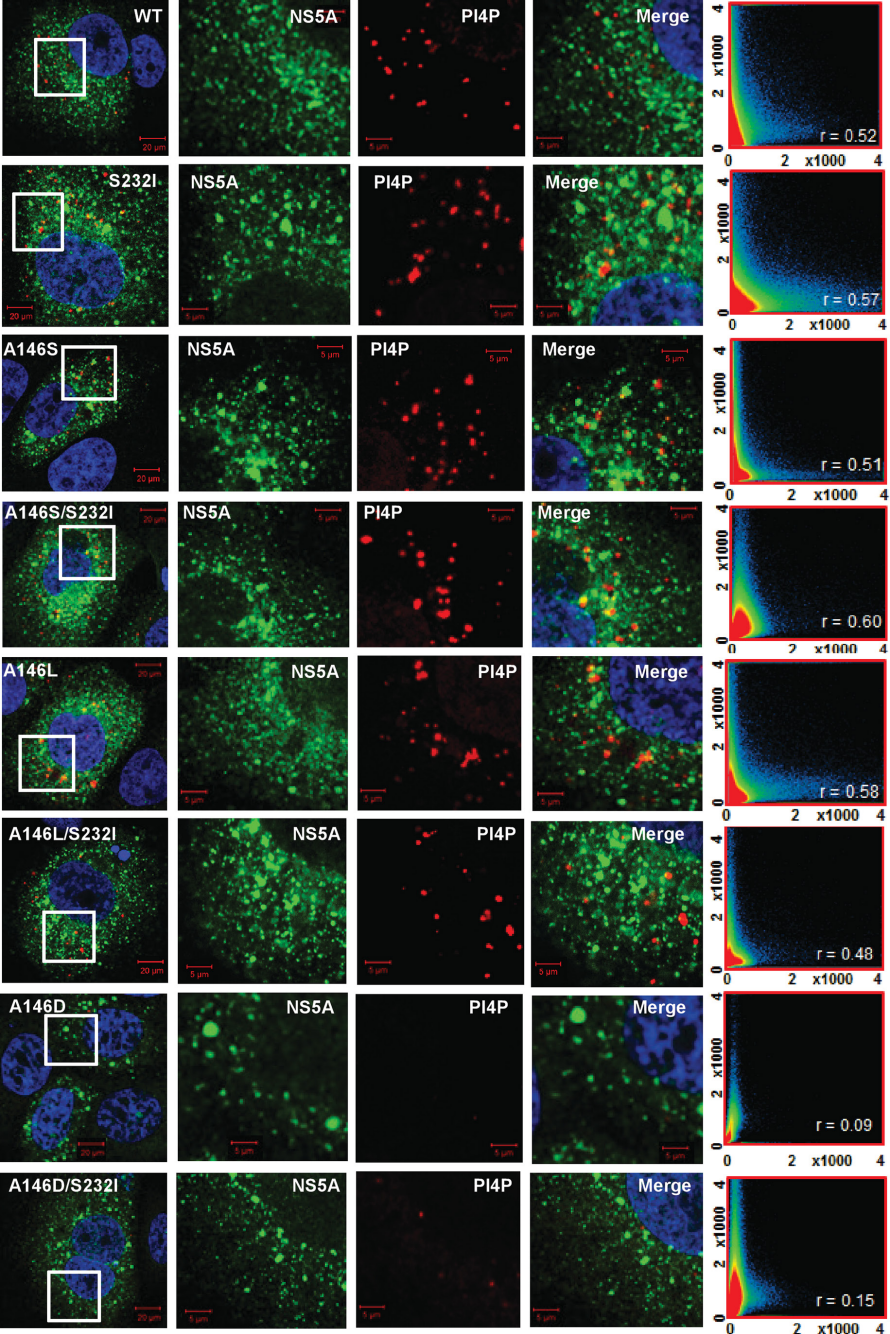
Colocalization of NS5A and the lipid phosphatidylinositol-4 phosphate (PI4P). Huh7 cells were electroporated with *in vitro* transcripts of the indicated SGRs, and seeded onto coverslips. At 48 h p.e. cells were fixed and immunostained for NS5A and PI4P lipids (mouse monoclonal), prior to imaging by confocal microscopy. White boxes indicate the area expanded in the right-hand panels. Colocalization scatter plots are of a 200×200 µm area containing >10 cells, and show the correlation of absolute values. The correlation value, *r*, is shown for each image.

**Fig. 7.**
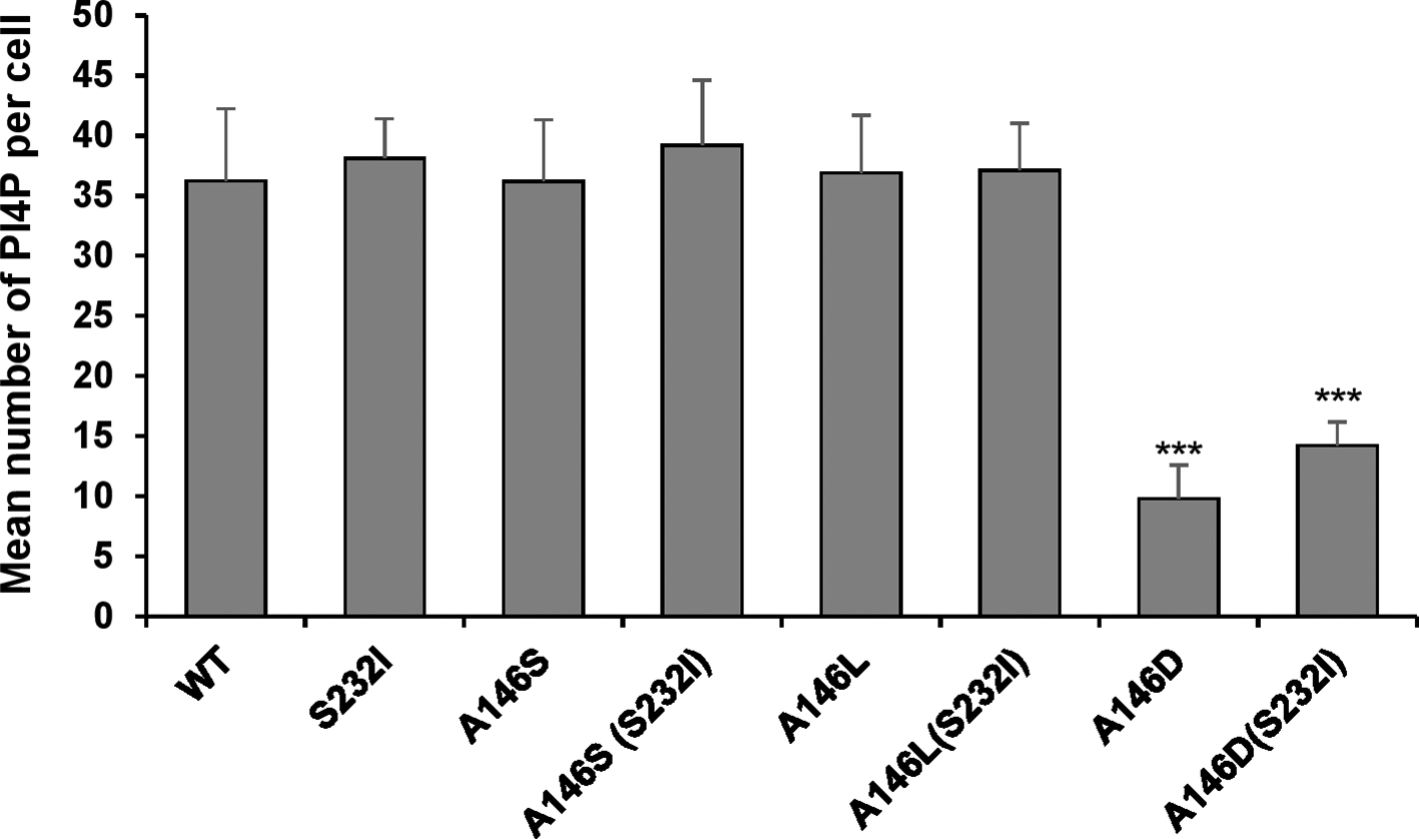
Quantification of NS5A:PI4P colocalization. Spatial data for PI4P lipids were determined from 12 cells for each SGR using the GDSC plug-in for Fiji; these data were used to determine the number of PI4P lipid punctae per cell.

Consistent with the 1-log reduction in the replication of A146D, there was also a reduction in the number of NS5A foci (data not shown). This was also apparent when SGR electroporated cells were co-stained for both NS5A and other replication complex components: NS3 (Fig. 5) and PI4P lipids (Fig. 6). For all the mutants there was a strong colocalization with NS3; this was expected, as the two proteins participate directly in the process of genome replication. PI4P is the product of PI4K activity, and NS5A has previously been reported to interact with, and activate, this cellular lipid kinase [12, 26]. The colocalization of NS5A and PI4P was determined, although interestingly this was not as pronounced as that observed for JFH-1 NS5A [25]; correlation values of ~0.5 were observed for the wild-type and most mutants (compared with ∼0.8 for JFH-1). Interestingly, for A146D and A146D/S232I both the number of PI4P punctae (Fig. 7) and the correlation values reduced significantly, consistent with a loss of colocalization. These data suggest that although A146D and A146D/S232I were able to replicate, they were impaired in their ability to recruit and activate PI4K. Of note, although S232I increased replication, it did not result in a concomitant increase in PI4P abundance.

### Residue 146 influences dimerization of Con1 NS5A domain I

**Fig. 8.**
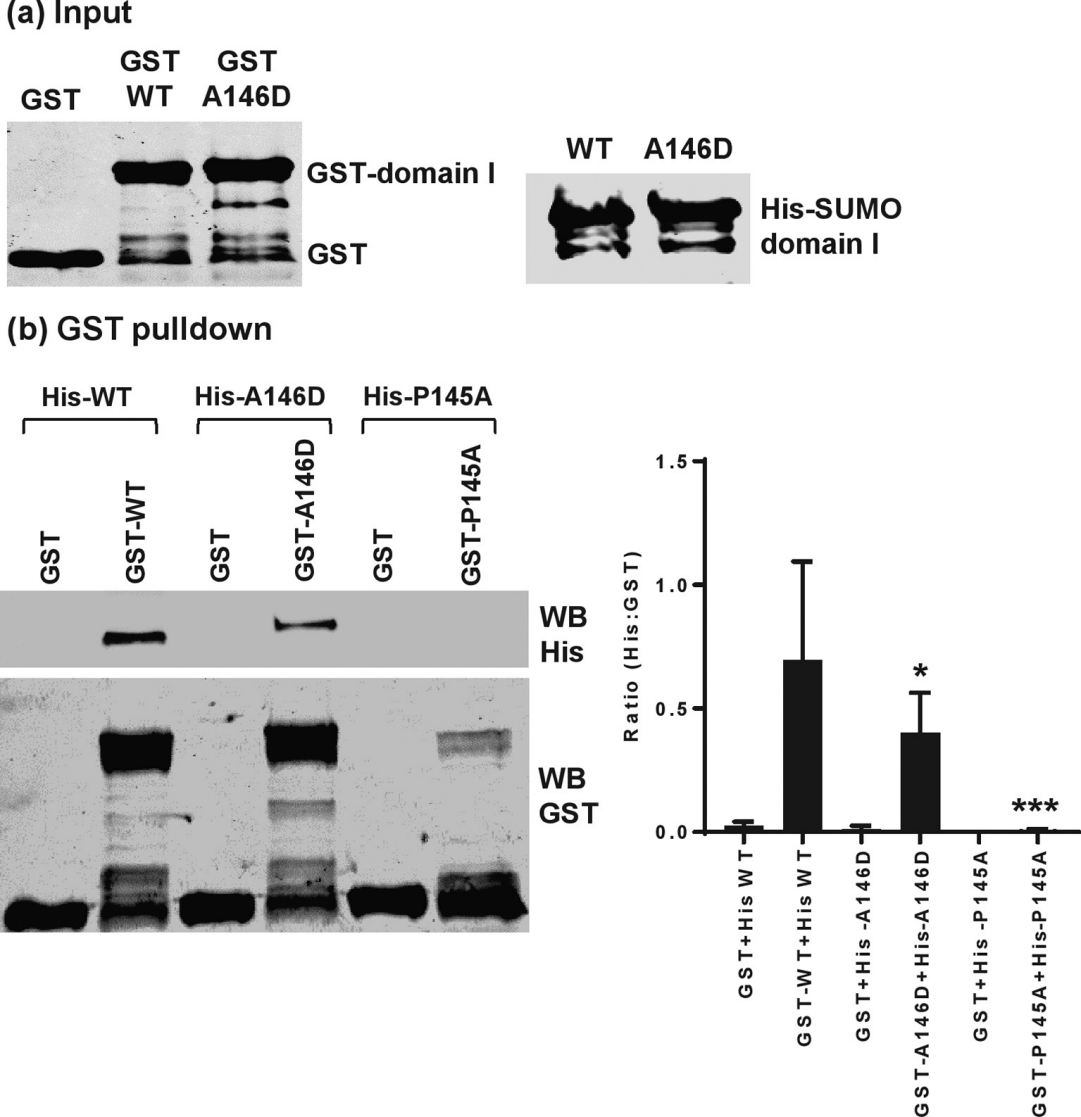
Residue 146 in domain I is involved in NS5A dimerization. (a) Input of GST and GST–domain I (35–215) (left), and His–SUMO–domain I (35–215) (right), analysed by Western blotting with anti-GST or anti-His antibodies. (b) His-tagged domain I proteins were used as prey in pulldown assays with GST or GST–domain I, with corresponding mutations as bait. Precipitated proteins were analysed by Western blotting using anti-His and anti-GST antibodies. The ratio of His : GST was calculated following quantification of Western blot signals using a Li-Cor Odyssey Sa infrared imaging system and represented graphically as a measure of the dimerization activity. These data were representative of three independent experiments using different batches of purified domain I proteins. Significant differences from the wild-type are denoted by * (*P*<0.01) or *** (*P*<0.001).

We [27], and others [28], have previously demonstrated that domain I of NS5A is able to dimerize *in vitro*. We also identified that residue P145 in JFH-1 NS5A was critical for this interaction, as a P145A mutation disrupted dimerization. Visual analysis of the ‘closed’ dimer form of genotype 1b NS5A domain I [6] revealed that both P145 and A146 were located in the dimer interface and this led us to ask whether residue 146 might also be involved in dimerization. To test this we expressed both Con1 wild-type and A146D domain I in *Escherichia coli,* and used GST-tagged domain I as bait to precipitate His-tagged domain I, as described previously [27]. Fig. 8 shows that the A146D mutation significantly reduced the ability of Con1 domain I to dimerize, although compared to the negative control (JFH-1 P145A), A146D still exhibited dimer formation. These data are consistent with the hypothesis that in the context of Con1 residue 146 could be involved in the formation of the closed dimer form of NS5A.

## Discussion

The results of this study demonstrate that in the context of the genotype 1b Con1 isolate, residue 146 of NS5A plays a role in the regulation of the genome replication, sub-cellular localization and dimerization of NS5A, with corresponding effects on NS5A hyperphosphorylation. The key observation is that both A146D and the previously characterized culture-adaptive mutation S232I exhibited a loss of NS5A hyperphosphorylation, yet, whereas A146D reduced replication, S232I, as expected, increased replication. This contrasts with previous studies on genotype 1b SGRs, where loss of hyperphosphorylation was associated with enhanced replication [19–21]. An explanation for these conflicting data is that, at least in the context of the Con1 SGR, hyperphosphorylation of NS5A does not directly regulate genome replication. In contrast, we believe that these results indicate that a loss of hyperphosphorylation is in fact a consequence of the perturbation of replication complex formation and localization.

Further evidence for this hypothesis comes from the observation that although the regulation of hyperphosphorylation by residue 146 appears to be conserved in Con1 and JFH-1, the loss of hyperphosphorylation did not have any functional consequences for JFH-1. Mutation of residue 146 had no effect on genome replication in the context of either an SGR or infectious virus, or the sub-cellular distribution of NS5A and other markers of replication complexes [8, 25]. In contrast, A146D in Con1 resulted in both a significant perinuclear restriction (Figs 3 and 4) and a reduction in the number of PI4P foci per cell (Figs 6 and 7), but this appears not to be related to hyperphosphorylation, as S232I had no effect on either parameter, yet also resulted in a loss of hyperphosphorylation (Fig. 2). Again, this points to the conclusion that loss of hyperphosphorylation is a consequence, rather than a cause, of these phenotypes.

The hyperphosphorylated form of NS5A is now generally accepted to be the result of the phosphorylation of multiple serine residues within LCSI, in particular S235 [17, 29]. In this regard, hyperphosphorylation can be directly disrupted by mutation of phosphoacceptor sites, and this is the mechanism by which S232I disrupts hyperphosphorylation. Sequential (also termed hierarchical) phosphorylation proceeds from S232 to S235 and S238 within LCSI, and S232I would thus be predicted to disrupt this process. Indeed, the use of phosphospecific antibodies has shown that S232A blocked phosphorylation of S235 and S238, whereas S232D rescued these phosphorylation events [30].

A key question is therefore how might residue 146 influence the phosphorylation status of NS5A? As discussed previously [8], in genotype 1b residue 146 lies at the interface of the closed dimer structure [6], and it is plausible that, if this dimer structure was also present in JFH-1, phosphorylation at this residue could disrupt the closed dimer and favour either a monomer or the ‘open’ dimer configuration [5]. Phosphorylation at residue 146 could therefore result in both dynamic and reversible effects on LCSI hyperphosphorylation by imparting a negative charge at that residue, however, clearly such an effect cannot occur in genotype 1b (or other genotypes lacking a phosphorylatable residue at 146). The observation that the A146D mutation was associated with a significant reduction in the dimerization of domain I is consistent with the hypothesis that residue 146 is present within the dimer interface and contributes to dimerization. A146D is a phosphomimetic mutation and thus introduces a negative charge at this residue, which is likely to lead to conformational rearrangements in the protein, influencing both dimerization and hyperphosphorylation, and the observation that neither A146S nor A146L disrupt hyperphosphorylation (Fig. 2b) is consistent with this hypothesis. Intriguingly, A146D appeared to exert a dominant replication phenotype, as in the context of A146D, S232I did not enhance replication as it did in a wild-type (A146) background. This is also consistent with the hypothesis that NS5A dimerization contributes to the establishment of a conformation competent for the initiation of a hierarchical phosphorylation cascade within LCSI. As further support for this, recent evidence has linked defects in dimerization to a loss of hyperphosphorylation [31], although it should be noted that in this study dimerization was determined by two-hybrid analysis and to date there has been no report of NS5A dimerization in HCV-infected cells.

Interpretation of the role of residue 146 is confounded by the fact that it is an alanine in the majority of HCV isolates, but in genotypes 1a, 1 c and 2a this residue is serine. As discussed above, the absence of a phosphorylatable residue at 146 in the vast majority of HCV isolates implies that the function cannot be directly affected by phosphorylation. The lack of phenotype for the Con1 A146S mutation, in comparison to the phosphomimetic A146D, implies that even when a phosphorylatable residue is present at 146, it is not a substrate for phosphorylation. Given that S146 phosphorylation was observed in JFH-1, this alludes to differences in the conformation of NS5A domain I between the two genotypes, such that residue 146 is not accessible to cellular kinases. This hypothesis is supported by the observation that the phosphomimetic mutation at residue 146 only decreased genome replication in the context of the Con1 isolate, and not in JFH-1. It is noteworthy that S146 phosphorylation per se is not incompatible with hyperphosphorylation, as pS146 was identified in both p56 and p58 species by mass spectrometry [18], even though this study also confirmed that the phosphomimetic S146D mutation was associated with a loss of hyperphosphorylation.

Taken together, our study reveals fundamental differences between HCV isolates with regard to both the structure of domain I and the regulation of NS5A function, and suggests that caution should be taken when extrapolating data obtained with the JFH-1 system, as these may not be generally applicable to all HCV genotypes. The availability of SGRs and infectious viruses derived from six of the seven genotypes [32, 33] will enable future comparative studies to address these questions. Our future efforts are focused on investigating the link between residue 146 and hyperphosphorylation in LCSI – we hope that a proteomic analysis comparing the wild-type with phosphorylation site mutants will reveal essential protein–protein interactions that can help to explain our observations.

## Methods

### Plasmids and mutagenesis

The CpG/UpA low luciferase SGR (SGR-luc-Con1) and the corresponding GND (NS5B polymerase defective) derivative were used throughout this study (a kind gift from Peter Simmonds, University of Oxford) [23]. Site-directed mutagenesis of NS5A was performed by generating overlapping PCR fragments between *Mlu*I and *Xho*I restriction sites (657-nt), carrying the desired nucleotide exchange, and then cloning into SGR-luc-Con1 via flanking *Mlu*I/*Xho*I sites and confirming by sequencing.

### Cell culture

Huh7.5 cells were cultured in Dulbecco's Modified Eagles Medium (DMEM) (Sigma) supplemented with 10 % foetal bovine serum (FBS), 100 IU penicillin ml^−1^, 100 µg streptomycin ml^−1^ and 1 % non-essential amino acids in a humidified incubator at 37 °C, 5 % CO_2_.

### Electroporation of SGR RNA constructs

The preparation of *in vitro* transcripts and their electroporation into Huh7 cells was conducted as described previously [8]. In brief, 4.5×10^6^ Huh7.5 cells in diethylpyrocarbonate (DEPC) treated PBS were electroporated with 3 µg of *in vitro* RNA transcripts using a square wave protocol, 260 V and 25 ms pulse. Subsequently, cells were resuspended in complete DMEM media and seeded at 1×10^4^ cells cm^−2^ culture area, into either 12- or 6-well dishes.

### Transient replication luciferase assays

Wild-type and mutant SGR-luc-Con1 RNAs were electroporated into Huh7.5 cells, seeded into 96-well format and cell extracts were prepared at 4, 24, 48, and 72 h p.e. Luciferase activity was determined using the luciferase assay system (Promega) and a Biotrace M3 luminometer (Biotrace Ltd). Data points were obtained in triplicate, and experiments were repeated at least three times; representative data are given for each experiment.

### SDS-PAGE/Western blot

Cells were washed twice in PBS, lysed in 1× Glasgow lysis buffer [GLB; 1 % (v/v) Triton X-100, 120 mM KCl, 30 mM NaCl, 5 mM MgCl_2_, 10 % (v/v) glycerol, 10 mM PIPES-NaOH, pH 7.2, with protease and phosphatase inhibitors] and harvested by centrifugation (2800 ***g***, 10 min, 4 °C) before determining and normalizing protein concentration by BCA assay (Pierce). Following separation by SDS-PAGE, proteins were transferred to a polyvinylidene difluoride (PVDF) membrane and blocked in 50 % (v/v) Odyssey blocking (OB) buffer (LI-COR) in PBS. The membrane was incubated with primary antibodies overnight at 4 °C, followed by secondary antibodies for 2 h at room temperature, both prepared in 50 % OB buffer. The primary antibodies used were: anti-NS5A (sheep), 1 : 5000 [34]; anti-NS3 (mouse), 1 : 2 000; and anti-glyceraldehyde-3-phosphate dehydrogenase (GAPDH, mouse), 1 : 20 000 (Sigma). The secondary antibodies were anti-goat (800 nm) or anti-mouse (700 nm), used at 1 : 10 000 prior to imaging fluorescence using a LI-COR Odyssey Sa infrared imaging system. Quantification of fluorescently labelled Western blots were carried out using Image Studio v3.1 (LI-COR) using a background subtraction method.

### Immunofluorescence and confocal microscopy

Cells were washed with PBS before fixation for 20 min in 4 % (w/v) PFA, and they were subsequently permeabilized in 0.1 % (v/v) Triton X-100, PBS and blocked with PBS-T, 5 % (w/v) BSA before immunostaining with the denoted antibody. The primary antibodies used were: anti-NS5A (sheep), 1 : 1,000; anti-NS3 (mouse), 1 : 350; and anti-PI4P (mouse), 1 : 100 (Echelon). Various fluorescently conjugated secondary antibodies were used at 1 : 1000 (Life Technology). LDs were stained using the BODIPY (558/568)-C12 dye at 1 : 5000 (Life Technology), added at the same time as the fluorescent secondary antibody. Nuclei were counterstained with 4′,6′-diamidino-2-phenylindole (DAPI). Confocal microscopy images were acquired on a Carl Zeiss LSM 880 inverted microscope, and post-acquisition analysis was conducted using Zen software (Zen version 2012 black edition 8.0, Zeiss, Germany).

### Quantification of LDs

For the quantification of LD spatial arrangement, images were acquired with the same acquisition parameters, but with variable gain to ensure correct exposure. The spatial coordinates of LDs were determined using the FindFoci function of the GDSC plugin for Fiji, with the nuclear envelope being manually outlined (utilizing the DAPI staining as reference) and coordinates generated by Fiji. The distance from each LD to the nuclear envelope was then determined using trigonometry. LD spatial distribution data were generated for 12 randomly selected cells for each replicon variant and the data were combined into a box-and-whisker plot.

### GST-pulldown assay

The construction and purification of GST–domain I (GST-DI) and His–SUMO–domain I (His-SUMO-DI) were described previously [27]. Briefly, the coding sequence for either WT or mutant (A146D) domain 1 (amino acids 35 to 215) of Con1 (genotype 1b) NS5A was amplified by PCR (and cloned into either pGEX6P-2 or pET28a-SUMO vectors to allow the expression of domain I N-terminally fused to either GST or a His-SUMO affinity tag. After purification, GST-DI and His-SUMO-DI were dialyzed into buffer (50 mM Tris-HCl, pH 7.5, 100 mM NaCl, 5 mM MgCl_2_, 10 % glycerol, 0.5 % NP-40). A GST pulldown assay was performed as described previously [28]. Briefly, 10 µg of GST or GST fusion proteins were mixed with 5 µg of His-SUMO-DI in binding buffer containing 20 mM Tris-HCl, pH 7.2, 0.5 M NaCl, 200 mM KCl and 1 % NP-40 for 3 h at 4 °C on a rotating platform. Then the mixture was added to glutathione beads and incubated overnight at 4 °C. After washes using binding buffer, bound material was eluted with 50 µL of SDS sample buffer, heated for 10 min at 95 °C and separated by SDS-PAGE for Western blot analysis using anti-GST and anti-His antibodies.

### Statistics

Datasets were analysed using Student’s *t* -test assuming a two-tailed, unequal variance to determine statistical difference from the wild-type. *n*=3 or greater throughout and *P<0.05, **P<0.01, ***P<0.001.
